# Supplementary material for: Non-Specific Elevated Serum Free Fatty Acids in Lung Cancer Patients: Nutritional or Pathological?
Source: Nutrients. 2024 Aug 28;16(17):2884. doi: 10.3390/nu16172884 (PMC11396813; doi:10.3390/nu16172884)
Supplement: Supplementary file 1 [file nutrients-16-02884-s001.zip › nutrients-3164110-supplementary.pdf]

Supplemental tables and figures

Table S1. Food Frequency Questionnaire (for all age 12 and older)

Food Name		Eat/drink or not<br>1 Yes<br>0 No	Frequency (answer only one of these)				Average amount for each time (grams or ml)
			Times per day	Times per week	Times per month	Times per year	
**Staple Food**							
1	Rice (cooked rice/rice flour)						gram
2	Wheat products (white bread, steamed bread/noodles/dumplings), non-fried, excluding instant noodles						gram
3	Whole grains (whole wheat breads, buckwheat)						gram
4	Deep-fried wheat products (fried bread stick, fried pancake, fried dough twist)						gram
5	Instant noodles						gram
6	Sweet potatoes						gram
7	Potato/taro/yam						gram
8	Coarse grains (corn, millet, sorghum, barley, rye)						gram
**Beans**							
9	Dried soybean (yellow, green or black)						gram
10	Soybean milk						ml
11	Soybean powder						gram
12	Tofu, soybean milk films						gram
13	All other beans (mung bean /red bean/kidney bean)						gram
**Vegetables**							
14	Fresh legumes (peas, snow peas, snap beans, yard long beans)						gram
15	Tomato						gram
16	Pepper (red, green, chili, etc.)						gram
17	Carrots						gram
18	Cucurbits vegetables (cucumber/pumpkin/summer squash, gourds)						gram
19	Green leafy `spinach/rape vegetable/water spinach/bok choy)						gram
20	Chinese cabbage and other leafy vegetables (cabbage/celery/lettuce, etc.)						gram
21	Cruciferous vegetables (Broccoli, cauliflower)						gram
22	Other fresh or frozen vegetables (bitter melon/lettuce stem/radish/water chestnut / bamboo shoots), excluding canned, dried, fermented, or pickled						gram
23	Allium (Garlic stalk/chives/Shallot/onion)						gram

24	Fungi and algae (mushrooms/seaweed)						gram
25	Dried vegetable (dehydrated vegetables, dried lily, not salted/pickled/fermented)						gram
26	Pickled vegetables (pickled cabbage, pickled mustard tuber, beans, etc.)						gram
27	Fermented foods (fermented bean curd/paste, douchi, excluding fermented milk products, beer, soy sauce, and vinegar)						gram
**Fruits**							
28	Orange and citrus (tangerine/apricot/persimmon/mango/papaya/lemons)						gram
29	Watermelon/honeydew melon/other melons						gram
30	All other fresh/frozen fruits (apples, berries, bananas, kiwi, etc.)						gram
31	All other dried fruits excluding preserved fruits (apples, berries, bananas, jujube, etc.)						gram
32	Preserved fruits including canned or sweetened						gram
**Milk**							
33	Whole milk						ml
34	Low-fat milk/nonfat milk						ml
35	Whole milk powder						gram
36	Low-fat/non-fat milk powder						gram
37	Yogurt						gram
38	Cheese						gram
39	Ice cream						gram
**Meats**							
40	Chicken, duck, goose, pigeon, quail (fried/non-fried)						gram
41	Lean pork						gram
42	Fatty pork						gram
43	Beef/ Lamb/mutton/other non-processed meats						gram
44	Processed Meat products (sausage/ham/luncheon meat)						gram
45	Avian and livestock viscera						gram
46	Fish—all types ocean and fresh water						gram
47	Shrimp and crab or other sea foods						gram
**Eggs**							
48	Fresh eggs (chicken/duck/quail egg)						gram
49	preserved eggs						gram
**Snacks**							
50	Salty soda cracker, salty mooncake						gram

51	Sweetened cookies, biscuits, cakes, pastries, and mooncake						gram
52	Nuts (peanut/ sunflower seeds/ pumpkin seeds/watermelon seeds/other seeds)						gram
53	Chocolate						gram
54	Potato chips/French fries/other fried snacks						gram
**Alcohol and beverages**							
55	Liquor (all types)						gram
56	Grape wine/yellow rice wine/rice wine						gram
57	Beer						gram
58	Water, plain or carbonated with nothing added						ml
59	Artificially sweetened beverages including sodas, commercial teas						ml
60	Sugar-sweetened beverages e.g. soft drinks, fruit-flavored drinks, milk-flavored drinks, sugared waters and commercial teas, sports or energy drinks						ml
61	Tea						ml
62	Coffee with/without sugar and/or milk						ml
63	100% fruit juice						ml

Table S2. Multivariate logistic regression analyses of serum free fatty acids (FFAs) between lung cancer patients and healthy controls stratified by smoking status.

FFAs	Smokers (n=224)			Non-smokers (n=622)		
	OR	95% CI	p value	OR	95% CI	p value
C12.0	1.097	0.925-1.362	.329	1.112	1.038-1.201	.004
C12.1	1.794	1.041-3.402	.051	1.093	0.971-1.306	.240
C14.0	1.122	1.023-1.240	.019	1.132	1.091-1.177	<.001
C14.1	3.346	1.517-8.213	.005	1.878	1.443-2.490	<.001
C15.0	1.311	0.779-2.294	.321	1.446	1.162-1.825	.001
C16.1	1.024	1.000-1.049	.053	1.053	1.040-1.067	<.001
C17.0	1.164	0.856-1.607	.341	1.859	1.546-2.261	<.001
C17.1	1.140	0.825-1.595	.432	1.978	1.635-2.424	<.001
C18.1	1.003	1.000-1.006	.032	1.006	1.004-1.007	<.001
C18.2	1.001	0.998-1.003	.585	1.007	1.006-1.009	<.001
C18.3 α	1.066	1.021-1.120	.007	1.126	1.097-1.157	<.001
C18.3 γ	2.025	1.380-3.153	.001	2.774	2.198-3.560	<.001
C20.1	1.151	0.988-1.362	.084	1.719	1.522-1.958	<.001
C20.2	1.248	0.983-1.604	.075	2.041	1.746-2.413	<.001
C20.3	1.951	1.355-2.926	.001	2.923	2.296-3.783	<.001
C20.4	1.111	0.998-1.244	.060	1.152	1.083-1.229	<.001
C20.5	1.295	0.679-2.583	.442	2.598	1.694-4.090	<.001
C22.1	2.228	1.263-4.672	.017	4.147	2.568-7.143	<.001
C22.4	1.731	1.109-2.791	.019	3.603	2.678-4.950	<.001
C22.5 ω3	1.323	0.849-2.101	.223	2.618	2.012-3.462	<.001
C22.5 ω6	3.701	1.529-9.628	.005	4.686	2.975-7.604	<.001
C22.6	1.020	0.930-1.121	.676	1.171	1.112-1.237	<.001
SFAs	1.085	1.012-1.171	.029	1.080	1.053-1.110	<.001
MUFAs	1.003	1.000-1.005	.030	1.005	1.004-1.007	<.001
PUFAs	1.001	0.999-1.003	.422	1.007	1.005-1.008	<.001
ω3 PUFAs	1.036	1.007-1.070	.022	1.083	1.064-1.104	<.001
ω6 PUFAs	1.001	0.999-1.003	.561	1.007	1.006-1.009	<.001
ω6 / ω3 PUFAs	0.966	0.914-1.008	.151	1.054	1.013-1.100	.014
Total FFAs	1.001	1.000-1.002	.118	1.003	1.002-1.004	<.001

Abbreviations: SFAs, saturated fatty acids; MUFAs, monounsaturated fatty acids; PUFAs, polyunsaturated fatty acids; ω3 PUFAs, ω3 polyunsaturated fatty acids; ω6 PUFAs, ω6 polyunsaturated fatty acids; ω6 / ω3 PUFAs, ratio of ω6 PUFAs to ω3 PUFAs.

Multivariate logistic regression models were adjusted for age, gender, BMI, and diabetes status.

Table S3. Multivariate logistic regression analyses of serum free fatty acids (FFAs) between lung cancer patients and healthy controls stratified by gender.

FFAs	Males (n=378)			Females (n=482)		
	OR	95% CI	p value	OR	95% CI	p value
C12.0	1.154	0.982-1.402	.112	1.101	1.027-1.189	.010
C12.1	1.809	1.182-2.907	.009	1.067	0.957-1.260	.328
C14.0	1.158	1.079-1.249	<.001	1.117	1.076-1.163	<.001
C14.1	3.112	1.738-5.917	<.001	1.782	1.349-2.406	<.001
C15.0	1.720	1.156-2.656	.010	1.333	1.057-1.703	.018
C16.1	1.041	1.022-1.062	<.001	1.049	1.035-1.064	<.001
C17.0	1.434	1.126-1.856	.005	1.799	1.467-2.237	<.001
C17.1	1.490	1.151-1.967	.003	1.886	1.533-2.355	<.001
C18.1	1.004	1.002-1.006	<.001	1.005	1.004-1.007	<.001
C18.2	1.003	1.001-1.005	.003	1.008	1.006-1.009	<.001
C18.3 α	1.093	1.055-1.136	<.001	1.120	1.088-1.155	<.001
C18.3 γ	2.119	1.574-2.940	<.001	2.830	2.166-3.777	<.001
C20.1	1.240	1.094-1.423	.001	1.819	1.575-2.127	<.001
C20.2	1.500	1.238-1.845	<.001	1.991	1.677-2.395	<.001
C20.3	2.100	1.572-2.876	<.001	2.951	2.239-3.975	<.001
C20.4	1.139	1.048-1.244	.003	1.120	1.045-1.204	.002
C20.5	1.959	1.163-3.418	.014	2.253	1.384-3.802	.002
C22.1	2.624	1.582-4.910	.001	4.184	2.465-7.726	<.001
C22.4	2.115	1.487-3.088	<.001	3.680	2.610-5.333	<.001
C22.5 ω3	1.749	1.247-2.505	.002	2.542	1.888-3.497	<.001
C22.5 ω6	4.130	2.139-8.356	<.001	4.176	2.525-7.184	<.001
C22.6	1.102	1.029-1.184	.006	1.145	1.080-1.217	<.001
SFAs	1.111	1.052-1.180	<.001	1.070	1.043-1.101	<.001
MUFAs	1.004	1.002-1.006	<.001	1.005	1.004-1.006	<.001
PUFAs	1.003	1.001-1.004	.001	1.007	1.005-1.009	<.001
ω3 PUFAs	1.059	1.034-1.086	<.001	1.077	1.056-1.100	<.001
ω6 PUFAs	1.003	1.001-1.005	.003	1.007	1.006-1.009	<.001
ω6 / ω3 PUFAs	0.970	0.926-1.008	.156	1.075	1.026-1.133	.004
Total FFAs	1.002	1.001-1.003	<.001	1.003	1.002-1.004	<.001

Abbreviations: SFAs, saturated fatty acids; MUFAs, monounsaturated fatty acids; PUFAs, polyunsaturated fatty acids; ω3 PUFAs, ω3 polyunsaturated fatty acids; ω6 PUFAs, ω6 polyunsaturated fatty acids; ω6 / ω3 PUFAs, ratio of ω6 PUFAs to ω3 PUFAs.

Multivariate logistic regression models were adjusted for age, BMI, smoking status, and diabetes status.

Table S4. Multivariate logistic regression analyses of serum free fatty acids (FFAs) between lung cancer patients and healthy controls stratified by age.

FFAs	Age ≤ 50 (n= 460)			Age > 50 (n=400)		
	OR	95% CI	p value	OR	95% CI	p value
C12.0	1.078	1.009-1.159	.033	1.382	1.134-1.723	.002
C12.1	1.027	0.924-1.170	.623	2.616	1.703-4.193	<.001
C14.0	1.118	1.074-1.166	<.001	1.160	1.087-1.244	<.001
C14.1	1.847	1.379-2.531	<.001	2.591	1.548-4.572	.001
C15.0	1.243	0.978-1.596	.080	1.889	1.292-2.879	.002
C16.1	1.056	1.041-1.073	<.001	1.033	1.017-1.052	<.001
C17.0	1.546	1.273-1.898	<.001	1.814	1.399-2.403	<.001
C17.1	1.775	1.445-2.212	<.001	1.645	1.273-2.171	<.001
C18.1	1.005	1.004-1.007	<.001	1.005	1.003-1.007	<.001
C18.2	1.008	1.006-1.010	<.001	1.003	1.001-1.004	.001
C18.3 α	1.147	1.110-1.189	<.001	1.069	1.038-1.104	<.001
C18.3 γ	2.739	2.095-3.657	<.001	2.321	1.729-3.217	<.001
C20.1	1.713	1.488-1.997	<.001	1.335	1.175-1.537	<.001
C20.2	1.964	1.643-2.381	<.001	1.607	1.337-1.959	<.001
C20.3	2.909	2.192-3.952	<.001	2.264	1.706-3.078	<.001
C20.4	1.036	0.965-1.114	.327	1.294	1.181-1.428	<.001
C20.5	2.367	1.387-4.142	.002	2.005	1.245-3.375	.006
C22.1	3.226	1.949-5.835	<.001	3.515	2.016-6.843	<.001
C22.4	2.858	2.071-4.042	<.001	2.959	2.038-4.433	<.001
C22.5 ω3	2.648	1.923-3.730	<.001	1.837	1.352-2.552	<.001
C22.5 ω6	4.099	2.397-7.243	<.001	4.351	2.381-8.423	<.001
C22.6	1.189	1.112-1.277	<.001	1.084	1.022-1.154	.009
SFAs	1.067	1.039-1.098	<.001	1.126	1.069-1.192	<.001
MUFAs	1.005	1.003-1.006	<.001	1.004	1.002-1.006	<.001
PUFAs	1.007	1.006-1.009	<.001	1.003	1.001-1.004	<.001
ω3 PUFAs	1.097	1.072-1.124	<.001	1.045	1.024-1.068	<.001
ω6 PUFAs	1.008	1.006-1.010	<.001	1.003	1.001-1.005	.001
ω6 / ω3 PUFAs	1.062	1.012-1.118	.018	0.995	0.965-1.026	.737
Total FFAs	1.003	1.002-1.004	<.001	1.002	1.001-1.003	<.001

Abbreviations: SFAs, saturated fatty acids; MUFAs, monounsaturated fatty acids; PUFAs, polyunsaturated fatty acids; ω3 PUFAs, ω3 polyunsaturated fatty acids; ω6 PUFAs, ω6 polyunsaturated fatty acids; ω6 / ω3 PUFAs, ratio of ω6 PUFAs to ω3 PUFAs.

Multivariate logistic regression models were adjusted for age, gender, BMI, smoking status, and diabetes status.

Table S5. Multivariate logistic regression analyses of serum free fatty acids (FFAs) between lung cancer patients and healthy controls stratified by BMI.

FFAs	BMI < 24 (n= 494)			24 ≤ BMI < 28 (n=279)			BMI ≥ 28 (n=85)		
	OR	95% CI	p value	OR	95% CI	p value	OR	95% CI	p value
C12.0	1.076	1.003-1.163	.050	1.191	1.007-1.464	.065	1.327	0.999-1.928	.096
C12.1	1.062	0.954-1.242	.335	1.924	1.198-3.265	.010	1.795	0.702-4.876	.223
C14.0	1.109	1.068-1.156	<.001	1.182	1.093-1.286	<.001	1.166	1.026-1.353	.028
C14.1	1.765	1.317-2.420	<.001	2.913	1.560-5.820	.001	1.946	0.867-5.234	.136
C15.0	1.364	1.087-1.739	.009	1.414	0.859-2.405	.185	2.555	1.107-6.763	.037
C16.1	1.045	1.031-1.059	<.001	1.054	1.030-1.080	.000	1.047	1.004-1.097	.039
C17.0	1.691	1.383-2.096	<.001	1.763	1.289-2.471	.001	1.356	0.842-2.241	.212
C17.1	1.759	1.440-2.182	<.001	1.751	1.251-2.505	.001	1.724	0.981-3.267	.069
C18.1	1.005	1.003-1.006	<.001	1.006	1.004-1.009	<.001	1.005	1.001-1.009	.022
C18.2	1.006	1.004-1.008	<.001	1.005	1.003-1.008	<.001	1.004	0.999-1.008	.101
C18.3 α	1.103	1.074-1.136	<.001	1.139	1.086-1.200	<.001	1.116	1.031-1.222	.010
C18.3 γ	2.413	1.874-3.176	<.001	2.992	2.008-4.648	<.001	3.086	1.545-7.125	.004
C20.1	1.496	1.326-1.703	<.001	1.525	1.279-1.852	<.001	1.712	1.179-2.660	.009
C20.2	1.783	1.514-2.129	<.001	1.902	1.485-2.486	<.001	1.835	1.182-3.056	.011
C20.3	2.538	1.960-3.359	<.001	2.916	1.987-4.440	<.001	2.898	1.421-6.620	.006
C20.4	1.109	1.037-1.188	.003	1.211	1.086-1.359	.001	1.061	0.868-1.297	.553
C20.5	2.422	1.499-4.064	<.001	2.147	1.170-4.124	.017	0.873	0.182-3.966	.859
C22.1	2.947	1.867-4.980	<.001	8.667	3.110-28.759	<.001	2.362	0.910-7.824	.103
C22.4	3.061	2.214-4.346	<.001	3.053	1.934-5.019	<.001	2.826	1.234-7.285	.019
C22.5 ω3	2.333	1.754-3.168	<.001	2.324	1.514-3.703	<.001	1.536	0.633-3.800	.342
C22.5 ω6	3.979	2.424-6.775	<.001	6.342	2.761-15.788	<.001	4.954	1.093-26.178	.045
C22.6	1.142	1.078-1.214	<.001	1.146	1.056-1.249	.001	1.012	0.849-1.206	.889
SFAs	1.065	1.037-1.096	<.001	1.124	1.059-1.200	<.001	1.119	1.019-1.256	.033
MUFAs	1.004	1.003-1.006	<.001	1.006	1.003-1.008	<.001	1.005	1.001-1.009	.020
PUFAs	1.006	1.004-1.007	<.001	1.005	1.003-1.007	<.001	1.004	1.000-1.008	.084
ω3 PUFAs	1.069	1.049-1.091	<.001	1.089	1.055-1.127	<.001	1.057	1.001-1.120	.050
ω6 PUFAs	1.006	1.004-1.008	<.001	1.005	1.003-1.008	<.001	1.004	0.999-1.008	.102
ω6 / ω3 PUFAs	1.032	0.998-1.077	.102	0.971	0.910-1.028	.335	0.969	0.847-1.101	.637
Total FFAs	1.003	1.002-1.003	<.001	1.003	1.002-1.004	<.001	1.002	1.000-1.004	.032

Abbreviations: SFAs, saturated fatty acids; MUFAs, monounsaturated fatty acids; PUFAs, polyunsaturated fatty acids; ω3 PUFAs, ω3 polyunsaturated fatty acids; ω6 PUFAs, ω6 polyunsaturated fatty acids; ω6 / ω3 PUFAs, ratio of ω6 PUFAs to ω3 PUFAs.

Multivariate logistic regression models were adjusted for age, gender, BMI, smoking status, and diabetes status.

Table S6. lipid metabolism genes RNA-seq of tumors (N=346) vs normal tissues (N=401)

Gene ID	Gene	Expression in tissues		Log_2FC	*P* value	*FDR*
		Normal tissues	Tumors			
ENSG00000076555	ACACB	31.91 (25.80-39.40)	9.41 (5.98-14.76)	-1.588	2.18E-100	8.10E-99
ENSG00000115361	ACADL	12.48 (9.01-16.57)	1.47 (0.50-3.66)	-2.316	2.38E-100	8.10E-99
ENSG00000196616	ADH1B	268.70 (198.30-345.70)	22.91 (6.83-73.87)	-2.328	2.41E-97	5.46E-96
ENSG00000187758	ADH1A	11.25 (8.25-15.30)	1.25 (0.43-3.52)	-2.268	1.15E-96	1.95E-95
ENSG00000188921	HACD4	7.05 (5.75-8.32)	2.73 (1.76-3.88)	-1.249	5.08E-95	6.91E-94
ENSG00000165996	HACD1	10.95 (8.95-12.61)	4.42 (2.69-7.03)	-0.980	3.37E-69	3.82E-68
ENSG00000111275	ALDH2	250.20 (208.50-291.90)	124.75 (72.14-174.92)	-0.916	2.04E-67	1.98E-66
ENSG00000110090	CPT1A	45.82 (38.71-52.68)	26.16 (19.61-34.00)	-0.665	2.41E-66	2.05E-65
ENSG00000145284	SCD5	22.31 (18.10-26.03)	10.25 (6.53-15.12)	-0.868	1.26E-63	9.53E-63
ENSG00000155016	CYP2U1	6.78 (5.39-7.74)	3.95 (2.67-5.23)	-0.654	7.44E-51	5.06E-50
ENSG00000127884	ECHS1	32.46 (28.61-35.14)	40.28 (34.38-47.20)	0.431	6.03E-48	3.73E-47
ENSG00000167315	ACAA2	37.54 (31.64-42.17)	25.39 (16.80-32.38)	-0.505	6.06E-42	3.44E-41
ENSG00000074696	HACD3	30.20 (26.36-35.97)	41.66 (32.95-54.57)	0.542	2.76E-39	1.44E-38
ENSG00000068366	ACSL4	40.80 (33.45-52.20)	27.08 (18.99-37.12)	-0.530	4.01E-37	1.95E-36
ENSG00000075239	ACAT1	48.05 (39.73-54.25)	32.84 (24.33-42.50)	-0.414	8.55E-37	3.88E-36
ENSG00000151726	ACSL1	66.46 (55.16-78.28)	45.88 (32.18-60.20)	-0.447	2.22E-36	9.45E-36
ENSG00000137124	ALDH1B1	13.45 (11.62-16.62)	20.49 (15.07-28.27)	0.722	2.36E-36	9.45E-36
ENSG00000133835	HSD17B4	173.80 (150.60-199.40)	121.00 (85.02-164.45)	-0.431	4.10E-35	1.55E-34
ENSG00000116353	MECR	9.19 (7.85-10.52)	11.75 (9.49-14.86)	0.444	5.19E-33	1.86E-32
ENSG00000087008	ACOX3	10.51 (9.27-11.77)	7.86 (5.94-9.97)	-0.347	3.17E-32	1.08E-31
ENSG00000105607	GCDH	9.88 (8.72-10.95)	12.19 (9.78-15.04)	0.400	2.53E-29	8.19E-29
ENSG00000084754	HADHA	89.44 (80.06-98.32)	74.56 (64.78-85.98)	-0.220	6.78E-27	2.05E-26
ENSG00000151093	OXSM	3.22 (2.68-3.77)	3.97 (3.34-4.87)	0.357	6.94E-27	2.05E-26
ENSG00000072778	ACADVL	242.70 (196.30-309.70)	182.05 (138.65-241.25)	-0.440	1.65E-26	4.67E-26
ENSG00000099797	TECR	165.40 (152.60-178.10)	196.00 (162.20-244.23)	0.426	2.83E-26	7.71E-26
ENSG00000136881	BAAT	0.37 (0.23-0.70)	1.58 (0.38-5.94)	2.469	2.26E-24	5.92E-24
ENSG00000138796	HADH	31.92 (28.83-35.26)	39.39 (30.93-50.04)	0.397	1.32E-21	3.32E-21
ENSG00000167969	ECI1	19.33 (16.09-22.17)	24.57 (18.18-32.07)	0.475	1.56E-21	3.80E-21
ENSG00000099194	SCD	186.30 (150.00-239.20)	127.60 (77.65-190.65)	-0.328	5.42E-20	1.27E-19
ENSG00000169710	FASN	121.10 (89.05-158.90)	82.03 (57.99-118.85)	-0.387	8.92E-19	2.02E-18
ENSG00000060971	ACAA1	43.00 (39.34-46.92)	36.30 (26.84-44.65)	-0.244	4.13E-18	9.05E-18
ENSG00000176715	ACSF3	22.67 (19.92-25.94)	19.22 (16.04-23.59)	-0.228	2.67E-17	5.66E-17
ENSG00000149084	HSD17B12	60.21 (52.12-67.82)	52.10 (41.10-62.00)	-0.191	6.90E-14	1.42E-13
ENSG00000184227	ACOT1	9.00 (7.10-10.95)	7.01 (5.50-9.68)	-0.262	1.03E-12	2.07E-12
ENSG00000248144	ADH1C	6.32 (4.83-8.08)	3.49 (1.02-9.01)	1.219	1.35E-11	2.62E-11
ENSG00000119673	ACOT2	14.19 (11.94-16.91)	12.15 (9.17-15.42)	-0.187	2.11E-11	3.98E-11
ENSG00000198721	ECI2	19.98 (16.76-22.88)	16.76 (13.20-21.04)	-0.153	2.69E-10	4.94E-10
ENSG00000122971	ACADS	9.32 (7.71-10.76)	7.60 (5.88-9.97)	-0.120	4.48E-10	8.02E-10
ENSG00000157184	CPT2	16.05 (14.34-17.69)	17.96 (14.16-23.23)	0.297	4.69E-10	8.18E-10
ENSG00000113790	EHHADH	6.51 (5.35-7.71)	7.47 (5.78-10.18)	0.435	2.88E-09	4.90E-09
ENSG00000169169	CPT1C	3.88 (3.08-5.13)	3.08 (2.10-4.81)	0.064	4.93E-09	8.18E-09

ENSG00000161533	ACOX1	50.24 (45.98-55.11)	45.53 (37.44-54.79)	-0.093	2.55E-08	4.12E-08
ENSG00000100294	MCAT	6.42 (5.57-7.44)	7.16 (5.89-9.01)	0.241	3.81E-08	6.02E-08
ENSG00000072210	ALDH3A2	70.69 (62.18-81.08)	57.84 (41.45-85.96)	-0.094	2.42E-07	3.74E-07
ENSG00000145439	CBR4	2.92 (2.26-3.73)	3.38 (2.46-4.64)	0.255	1.25E-06	1.89E-06
ENSG00000164904	ALDH7A1	24.43 (19.91-28.32)	27.35 (19.03-39.71)	0.327	1.36E-06	2.01E-06
ENSG00000196177	ACADSB	12.59 (10.76-14.74)	10.50 (6.69-16.04)	-0.012	3.38E-06	4.89E-06
ENSG00000205560	CPT1B	3.10 (2.09-4.64)	3.94 (2.63-5.59)	0.199	4.73E-06	6.71E-06
ENSG00000066322	ELOVL1	56.20 (50.12-62.90)	60.58 (48.49-75.22)	0.180	5.11E-05	7.09E-05
ENSG00000164181	ELOVL7	3.15 (2.52-3.89)	2.61 (1.60-4.10)	-0.005	5.27E-05	7.17E-05
ENSG00000204228	HSD17B8	11.63 (10.29-13.39)	13.16 (9.50-18.85)	0.330	6.13E-05	8.17E-05
ENSG00000197894	ADH5	54.21 (47.03-61.20)	48.84 (39.17-60.62)	0.009	9.40E-05	1.23E-04
ENSG00000012660	ELOVL5	53.75 (47.48-62.15)	58.23 (46.84-72.81)	0.138	2.74E-04	3.51E-04
ENSG00000134824	FADS2	39.56 (29.58-51.00)	44.95 (27.05-83.13)	0.663	3.60E-04	4.54E-04
ENSG00000197142	ACSL5	39.12 (33.91-44.37)	45.43 (21.48-87.25)	0.689	0.0019	0.0023
ENSG00000120437	ACAT2	9.27 (7.50-11.33)	9.88 (7.13-15.22)	0.404	0.0031	0.0038
ENSG00000170522	ELOVL6	2.12 (1.67-2.84)	2.58 (1.31-5.26)	0.797	0.0036	0.0043
ENSG00000138029	HADHB	54.79 (49.14-60.28)	52.32 (45.69-60.33)	-0.015	0.0352	0.0412
ENSG00000117054	ACADM	26.21 (22.75-29.88)	25.00 (19.78-31.08)	-0.024	0.0383	0.0441
ENSG00000123983	ACSL3	39.28 (34.04-44.66)	40.12 (32.52-49.86)	0.126	0.0888	0.1006
ENSG00000097021	ACOT7	16.57 (14.02-20.40)	16.96 (12.82-24.62)	0.230	0.2652	0.2957
ENSG00000206527	HACD2	20.92 (17.45-25.62)	20.55 (16.27-25.60)	0.045	0.3734	0.4096
ENSG00000255154	HTD2	3.60 (2.96-4.28)	3.49 (2.80-4.27)	-0.012	0.3863	0.4169
ENSG00000143149	ALDH9A1	25.36 (21.36-29.36)	24.70 (19.52-31.44)	0.040	0.5218	0.5544
ENSG00000278540	ACACA	54.48 (46.39-63.01)	53.44 (40.84-71.66)	0.112	0.5745	0.6010
ENSG00000149485	FADS1	28.97 (23.71-34.67)	28.33 (19.07-42.77)	0.221	0.8981	0.9253
ENSG00000116171	SCP2	49.98 (42.98-57.67)	50.34 (35.22-66.64)	0.055	0.9405	0.9538
ENSG00000177465	ACOT4	3.53 (2.67-4.46)	3.50 (2.49-4.76)	0.066	0.9538	0.9538

A

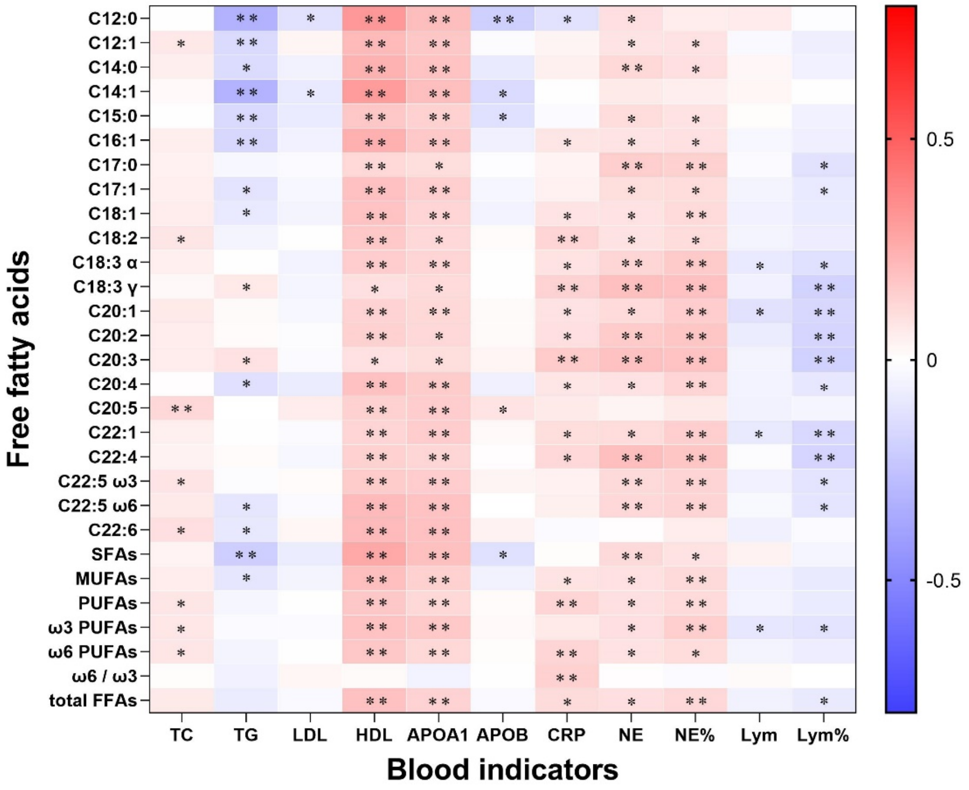
Free fatty acids
C12:0
C12:1
C14:0
C14:1
C15:0
C16:1
C17:0
C17:1
C18:1
C18:2
C18:3 α
C18:3 γ
C20:1
C20:2
C20:3
C20:4
C20:5
C22:1
C22:4
C22:5 ω3
C22:5 ω6
C22:6
SFAs
MUFAs
PUFAs
ω3 PUFAs
ω6 PUFAs
ω6 / ω3
total FFAs
TC
TG
LDL
HDL
APOA1
APOB
CRP
NE
NE%
Lym
Lym%
Blood indicators
0.5
0
-0.5

B

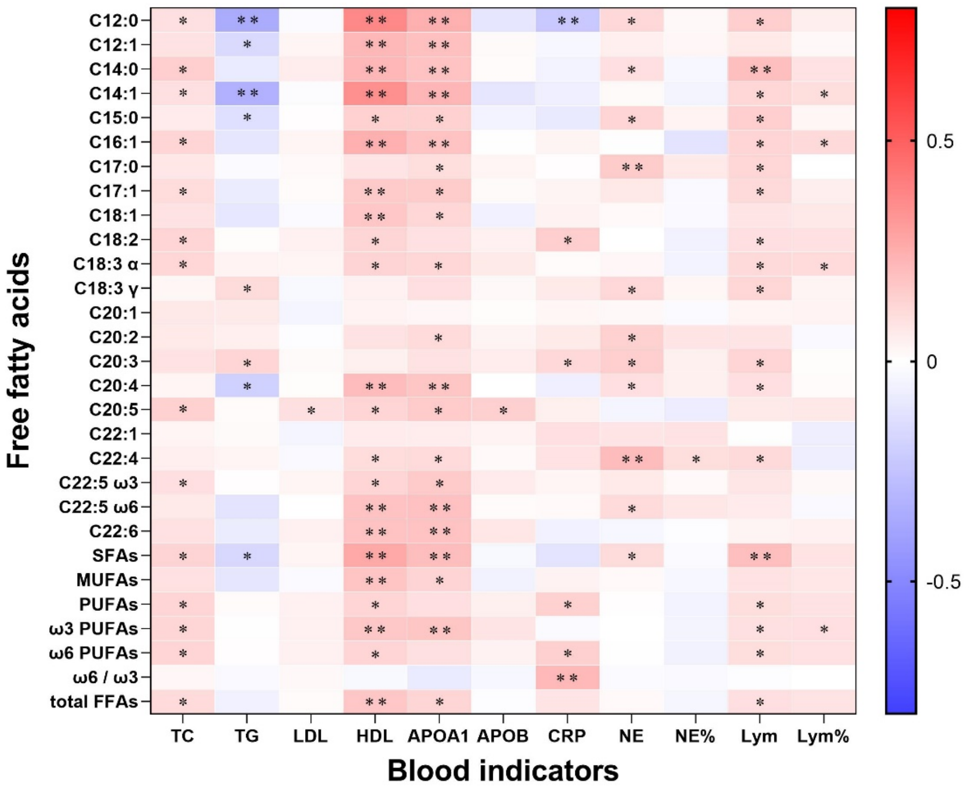
Free fatty acids
C12:0
C12:1
C14:0
C14:1
C15:0
C16:1
C17:0
C17:1
C18:1
C18:2
C18:3 α
C18:3 γ
C20:1
C20:2
C20:3
C20:4
C20:5
C22:1
C22:4
C22:5 ω3
C22:5 ω6
C22:6
SFAs
MUFAs
PUFAs
ω3 PUFAs
ω6 PUFAs
ω6 / ω3
total FFAs
TC
TG
LDL
HDL
APOA1
APOB
CRP
NE
NE%
Lym
Lym%
Blood indicators
0.5
0
-0.5

C

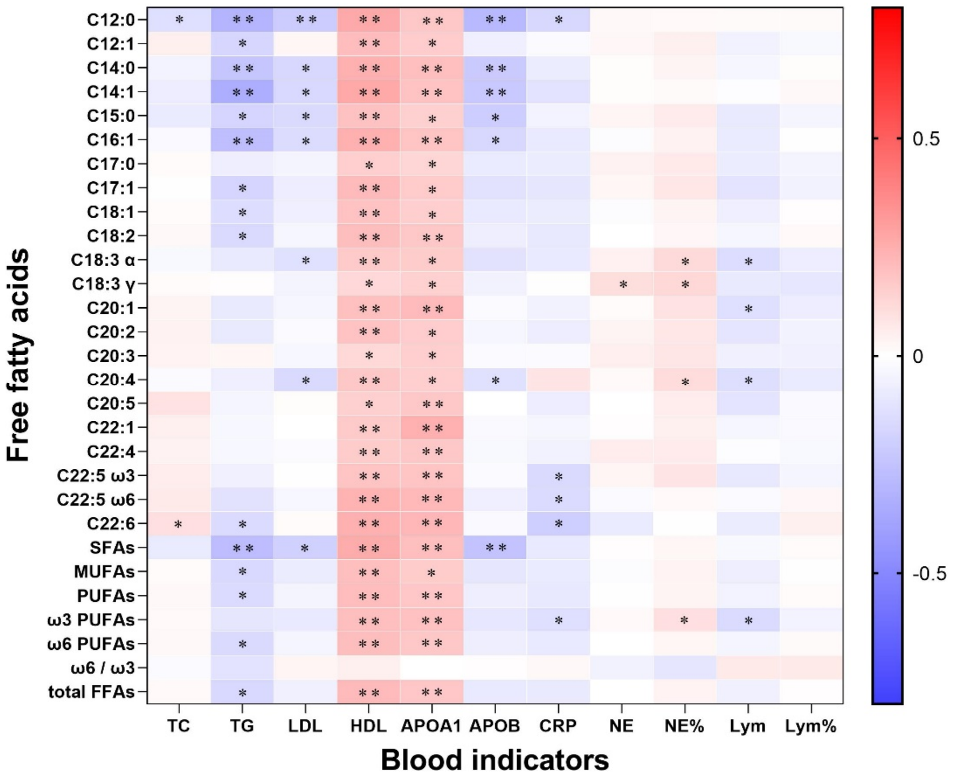

Figure S1. Spearman correlation between serum free fatty acids (FFAs) and blood indicators in (A) the whole study population (N=860); (B) the healthy control population (N=430); (C) the lung cancer patients (N=430).

Abbreviations: TC, total cholesterol; TG, Triglycerides; LDL, low-density lipoprotein cholesterol; HDL, high-density lipoprotein cholesterol; APOA1, apolipoprotein A1; APOB, apolipoprotein B; CRP, C-reactive protein; NE, neutrophil counts; NE%, neutrophil-leukocyte ratio; Lym, lymphocyte counts; Lym%, lymphocyte-leukocyte ratio.

* Indicated p<0.05. ** Indicated p<0.001.

Supplemental materials and methods

1. **Serum free fatty acids (FFAs) measurement**

1.1 Reagents and instrumentation

Twenty-seven reference standards and 8 deuterated internal standards (IS) of FFAs (purity≥98%) were purchased from NU-CHEK, Cmass and Aladdin. HPLC grade acetonitrile, 2-propanol, methanol, dichloromethane and formic acid were from ThermoFisher and TCI. Bovine serum albumin was from Sigma-Aldrich.

A SCIEX Triple Quad 7500 mass spectrometer (SCIEX, USA) coupled with Shimadzu 40D-X3 (Shimadzu, Japan) UPLC system was used for quantification of the FFAs in plasma samples. The FFAs were separated on an ACQUITY UPLC HSS T3 (2.6 μm, 100mm×3.0mm; Waters, USA) column.

1.2 Serum specimen preparation

Aliquot (20 μL) serum was added to a 180 μL internal standard working fluid in a 96-well plate, and shaken at 1450 rpm, 25°C for 10 min. Then the mixtures were centrifuged at 3076 g, 4°C for 10 min. An aliquot (100 μL) of supernatant was transferred into a clean 96-well plate. The plate was sealed with foil paper before testing.

1.3 LC-MS/MS method setting

An aliquot (0.5 μL) of the extracted sample on the plate was injected into the UPLC system. The mobile phase consisted of 0.01% formic acid in water (A), and acetonitrile/isopropyl (v/v = 50/50, B), with a flow rate of 200 μl/min. The elution gradient was: 0 - 1 min (75% B), 1 - 5 min (75% - 95% B), 5 - 9 min (95% B), 9 - 9.2 min (95% - 75% B), and 9.2 - 12 min (75% B). On each plate, the number of samples from cases and controls were the same, but the injection sequence was disordered. Before sampling, standard curve for every FFA ($R^2 > 0.99$) was constructed for absolute quantification of each analyte on this plate, and 4 known-concentration quality control samples were examined with each batch of the assay.

For each FFA to be detected, we first used a standard solution with a known concentration and obtained the mass spectrometry response values at different concentrations in the LC-MS/MS. We then constructed the corresponding standard curves with $R^2 > 0.99$. Thus, when measuring the concentration of FFAs in serum samples, we calculated the absolute concentration of each compound by comparing its mass spectrometry response to the standard curve. Therefore, the concentrations of the 27 FFAs were independently measured.

The mass spectrometer was operated with the following conditions: multiple reaction monitoring (MRM) scanning, mode ESI (-), capillary voltage 4.5 kV, cone voltage 55 V, and extractor voltage 4 V. The atomization and cone gas flow rates were 1000 and 150 L/H, respectively. The source temperature was 450°C, and the atomizing gas

temperature was 500℃. SCIEX OS software was used to collect the data with a mass range of 50 to 1000 Da. The scan time was set to 0.35 s, and the interscan delay was set to 0.02 s. Supplementary Table S5 showed 27 detected levels of serum free fatty acids in the study population.

1.4 Data processing

The LC-MS/MS raw data were analyzed by the SCIEX OS software. A list of the ion intensities of each detected peak was generated using the RT and m/z data pairs as the identifier for each ion. The resulting three-dimensional matrix contained arbitrarily assigned peak indices (retention time–m/z pairs), sample names (observations), and ion intensity information (variables). The internal standard and QC were used for data quality control (reproducibility).

Table S7. List of serum free fatty acids detected

FFAs	English name	CAS	Parent ion (m/z)	Daughter ion (m/z)	Internal standards
C12:0	dodecanoic acid	143-07-7	199.2	199.2	C14:0-D3
C12:1	11-dodecenoic	1289-45-8	197.2	197.2	C14:0-D3
C14:0	tetradecanoic acid	544-63-8	227.2	227.2	C14:0-D3
C14:1	tetradecenoic	544-64-9	225.2	225.2	C14:0-D3
C15:0	pentadecanoic acid	1002-84-2	241.2	241.2	C14:0-D3
C15:1	10-pentadecenoic acid	84743-29-3	239.2	239.2	C14:0-D3
C16:1	palmitoleic acid	373-49-9	253.2	253.2	C16:1-D13
C17:0	heptadecanoic acid	506-12-7	269.2	269.2	C16:1-D13
C17:1	10-heptadecenoic acid	29743-97-3	267.2	267.2	C16:1-D13
C18:1	oleic acid	593-39-5	281.3	281.3	C18:1-D17
C18:1 T	elaidic acid	593-40-8	281.4	281.4	C18:1-D17
C18:2	linoleic acid	60-33-3	279.2	279.2	C18:2-D4
C18:2 T	linoelaidic acid	506-21-8	279.4	279.4	C18:2-D4
C18:3 α	linolenic acid	463-40-1	277.2	277.2	C18:3-γ-D14
C18:3 γ	gamma linolenic acid	506-26-3	277.2	277.2	C18:3-γ-D14
C20:0	arachidic acid	506-30-9	311.4	311.4	C20:4-D11
C20:1	cis-11-eicosenoic acid	5561-99-9	309.3	309.3	C20:4-D11
C20:2	eicosa-11Z,14Z-dienoic Acid	2091-39-6	307.3	307.3	C20:4-D11
C20:3	eicosatrienoic acid	17046-59-2	305.3	305.3	C20:4-D11
C20:4	arachidonic acid	506-32-1	303.3	303.3	C20:4-D11
C20:5	eicosapentaenoic acid	10417-94-4	301.3	301.3	C20:5-D5
C22:0	behenic acid	112-85-6	339.6	339.6	C22:6-D5
C22:1	frucic acid	112-86-7	337.4	337.4	C22:6-D5
C22:4	docosatetraenoic acid	28874-58-0	331.4	331.4	C22:6-D5
C22:5 ω3	docosapentaenoic acid (cis-7,10,13,16,19)	24880-45-3	329.4	329.4	C22:6-D5
C22:5 ω6	4Z,7Z,10Z,13Z,16Z-	25182-74-5	329.4	329.4	C22:6-D5

	docosapentaenoic acid all-cis-4,7,10,13,16-DPA				
C22:6	cis-4,7,10,13,16,19-Docosahexaenoic acid	6217-54-5	327.3	327.3	C22:6-D5

2 RNA-sequencing of lung tumors and normal tissues

2.1 Cohorts and Clinical Specimens

A total of 417 patients with confirmed diagnosis of NSCLC were recruited from the Second Affiliated Hospital (SAH), Zhejiang University (Hangzhou, China) from February 2013 to June 2022. Matched tumor and normal tissue samples were collected from these patients who had not received previous systemic therapy and undergone surgical resection. Informed consent was obtained from each patient and approved by the Institutional Review Board of SAH. The demographic characteristics were abstracted from medical chart records and were shown in Supplementary Table S2.

All specimens were cut fresh from surgically resected samples and immediately snapped frozen in liquid nitrogen until placed in a -80°C freezer for storage. Genomic DNA and total RNA samples were extracted from frozen tissues using the DNA/RNA AllPrep Kit (Qiagen). DNA was quantified by the Qubit (Invitrogen) and DNA integrity was assessed by TapeStation (Agilent Technologies). Total amounts and integrity of RNA were assessed using the RNA Nano 6000 Assay Kit of the Bioanalyzer 2100 system (Agilent Technologies, CA, USA).

Table S8. Demographic characteristics of population

Variable	Normal (*N*=401)	Tumor (*N*=346)
Age, mean±sd	67.78±9.41	67.43±9.24
Gender, n (%)		
Male	228 (56.86)	193 (55.78)
Female	173 (43.14)	153 (44.22)
Smoking, n (%)		
Never	259 (64.59)	219 (63.29)
Current/Former	142 (35.41)	127 (36.71)
Pathologic stage, n (%)		
I	238 (59.35)	200 (57.80)
II	59 (14.71)	49 (14.16)
III	99 (24.69)	91 (26.30)
IV	2 (0.50)	2 (0.58)
NA	3 (0.75)	4 (1.16)
Clinical T stage, n(%)		
T1	232 (57.85)	198 (57.23)
T2	128 (31.92)	109 (31.50)
T3	23 (5.74)	17 (4.91)

T4	15 (3.74)	18 (5.20)
Tis	1 (0.25)	1 (0.29)
NA	2 (0.50)	3 (0.87)
Clinical N stage, n(%)		
N0	274 (68.33)	234 (67.63)
N1	40 (9.97)	34 (9.83)
N2	86 (21.45)	76 (21.96)
NA	1 (0.25)	2 (0.58)
Clinical M stage, n(%)		
M0	397 (99.00)	341 (98.55)
M1	2 (0.50)	2 (0.58)
NA	2 (0.50)	3 (0.87)
Death		
Yes	84 (20.95)	74 (21.39)
No	317 (79.05)	272 (78.61)
First progression		
Yes	147 (36.66)	127 (36.71)
No	254 (63.34)	219 (63.29)

2.2 RNA Sequencing and quality control

Ribosomal RNA was removed using MGIEasy 32 RXN (MGIEasy, China). Subsequently, the sequencing libraries suitable for paired-ended sequencing were generated using MGIEasy Total RNA Library Prep Kit (MGIEasy, China). RNA samples were pooled and sequenced on the MGI HiSeq platform (BGI, Shenzhen) and 150 bp paired-end reads were generated.

The sequencing quality of each sample was assessed using FastQC[1]. Picard Tools CheckFingerprint (Broad Institute, Cambridge, MA)[2] was used for quantifying sample-relatedness and detecting incorrectly paired sequencing datasets from different donors. rRNA reads were detected and removed using RiboDetector[3]. Reads were trimmed for adapter sequences and low-quality bases using Cutadapt[4] prior to alignment against the human reference genome (GRCh38/hg38), using STAR[5]. We only included samples with a depth of coverage greater than 20 million mapped paired-end reads. The mean library size was 10G. Transcript expression was quantified using the Isofox_v1.6.1[6] to generate count and Transcript Per Million (TPM). Isofox excludes genes from GC bias calculations and to determine a normalisation factor for "adjusted TPM". For any given sample, AdjustedTPM = rawTPM x constant with the constant determined by the normalisation. Batch

---

1 FastQC: A Quality Control Tool for High Throughput Sequence Data.

2 Detecting sample swaps in diverse NGS data types using linkage disequilibrium

3 Deng ZL, Münch PC, Mreches R, McHardy AC. Rapid and accurate detection of ribosomal RNA sequences using deep learning. Nucleic Acids Research. 2022.

4 Cutadapt removes adapter sequences from high-throughput sequencing reads.

5 STAR: ultrafast universal RNA-seq aligner.

6 https://github.com/hartwigmedical/hmftools/tree/master/isofox

correction was performed using the covariate from the edgeR package. An expression filter was applied, keeping genes with an expression value of at least 1 TPM in at least 20% (150/747) of samples in the dataset. In total, 12,816 genes were filtered out of the 39,226 unique genes.

2.3 Differential expression genes evaluation

The negative binomial generalized linear model was implemented to evaluate differential expression genes using R package edgeR. We adjusted potential batch effects. Significant changes were defined at FDR < 0.05 and logFC cutoff = mean(abs(logFC)) + 2*sd(abs(logFC))).

3 Missing Data Imputation

For the imputation of missing data: BMI (2), smoking status (14), CEA (75), CA125 (76), CA199 (75), NSE (169) in the construction of diagnostic models, the mice package was applied (method = PMM, m = 5, seed = 12345).

Table S9. List of candidate genes in the lipid metabolism pathways

Gene	ENSG ID	Affiliated pathway in KEGG
ACAA1	ENSG00000060971	hsa00071, hsa01040, hsa01212
ACAA2	ENSG00000167315	hsa00062, hsa00071, hsa01212
ACACA	ENSG00000278540	hsa00061, hsa01212
ACACB	ENSG00000076555	hsa00061
ACADL	ENSG00000115361	hsa00071, hsa01212
ACADM	ENSG00000117054	hsa00071, hsa01212
ACADS	ENSG00000122971	hsa00071, hsa01212
ACADSB	ENSG00000196177	hsa00071, hsa01212
ACADVL	ENSG00000072778	hsa00071, hsa01212
ACAT1	ENSG00000075239	hsa00071, hsa01212
ACAT2	ENSG00000120437	hsa00071, hsa01212
ACOT1	ENSG00000184227	hsa00062, hsa01040
ACOT2	ENSG00000119673	hsa00062, hsa01040
ACOT4	ENSG00000177465	hsa00062, hsa01040
ACOT7	ENSG00000097021	hsa00062, hsa01040
ACOX1	ENSG00000161533	hsa00071, hsa01040, hsa01212
ACOX3	ENSG00000087008	hsa00071, hsa01040, hsa01212
ACSBG1	ENSG00000103740	hsa00061, hsa00071, hsa01212
ACSBG2	ENSG00000130377	hsa00061, hsa00071, hsa01212
ACSF3	ENSG00000176715	hsa00061, hsa01212
ACSL1	ENSG00000151726	hsa00061, hsa00071, hsa01212
ACSL3	ENSG00000123983	hsa00061, hsa00071, hsa01212
ACSL4	ENSG00000068366	hsa00061, hsa00071, hsa01212
ACSL5	ENSG00000197142	hsa00061, hsa00071, hsa01212
ACSL6	ENSG00000164398	hsa00061, hsa00071, hsa01212
ADH1A	ENSG00000187758	hsa00071
ADH1B	ENSG00000196616	hsa00071
ADH1C	ENSG00000248144	hsa00071
ADH4	ENSG00000198099	hsa00071
ADH5	ENSG00000197894	hsa00071
ADH6	ENSG00000172955	hsa00071
ADH7	ENSG00000196344	hsa00071
ALDH1B1	ENSG00000137124	hsa00071
ALDH2	ENSG00000111275	hsa00071
ALDH3A2	ENSG00000072210	hsa00071
ALDH7A1	ENSG00000164904	hsa00071
ALDH9A1	ENSG00000143149	hsa00071
BAAT	ENSG00000136881	hsa01040
CBR4	ENSG00000145439	hsa00061, hsa01212
CPT1A	ENSG00000110090	hsa00071, hsa01212
CPT1B	ENSG00000205560	hsa00071, hsa01212

CPT1C	ENSG00000169169	hsa00071, hsa01212
CPT2	ENSG00000157184	hsa00071, hsa01212
CYP2U1	ENSG00000155016	hsa00071
ECHS1	ENSG00000127884	hsa00062, hsa00071, hsa01212
ECI1	ENSG00000167969	hsa00071
ECI2	ENSG00000198721	hsa00071
EHHADH	ENSG00000113790	hsa00071, hsa01212
ELOVL1	ENSG00000066322	hsa00062, hsa01040, hsa01212
ELOVL2	ENSG00000197977	hsa00062, hsa01040, hsa01212
ELOVL3	ENSG00000119915	hsa00062, hsa01040, hsa01212
ELOVL4	ENSG00000118402	hsa00062, hsa01040, hsa01212
ELOVL5	ENSG00000012660	hsa00062, hsa01040, hsa01212
ELOVL6	ENSG00000170522	hsa00062, hsa01040, hsa01212
ELOVL7	ENSG00000164181	hsa00062, hsa01040, hsa01212
FADS1	ENSG00000149485	hsa01040, hsa01212
FADS2	ENSG00000134824	hsa01040, hsa01212
FASN	ENSG00000169710	hsa00061, hsa01212
GCDH	ENSG00000105607	hsa00071
HACD1	ENSG00000165996	hsa00062, hsa01040, hsa01212
HACD2	ENSG00000206527	hsa00062, hsa01040, hsa01212
HACD3	ENSG00000074696	hsa00062, hsa01040, hsa01212
HACD4	ENSG00000188921	hsa00062, hsa01040, hsa01212
HADH	ENSG00000138796	hsa00062, hsa00071, hsa01212
HADHA	ENSG00000084754	hsa00062, hsa00071, hsa01212
HADHB	ENSG00000138029	hsa00062, hsa00071, hsa01212
HSD17B12	ENSG00000149084	hsa00062, hsa01040, hsa01212
HSD17B4	ENSG00000133835	hsa01040, hsa01212
HSD17B8	ENSG00000204228	hsa00061, hsa01212
HTD2	ENSG00000255154	hsa00061, hsa01212
MCAT	ENSG00000100294	hsa00061, hsa01212
MECR	ENSG00000116353	hsa00061, hsa00062, hsa01212
OLAH	ENSG00000152463	hsa00061
OXSM	ENSG00000151093	hsa00061, hsa01212
SCD	ENSG00000099194	hsa01040, hsa01212
SCD5	ENSG00000145284	hsa01040, hsa01212
SCP2	ENSG00000116171	hsa01040, hsa01212
TECR	ENSG00000099797	hsa00062, hsa01040, hsa01212

Pathways in KEGG:
hsa00061: fatty acid biosynthesis;
hsa00062: fatty acid elongation;
hsa00071: fatty acid degradation;
hsa01040: biosynthesis of unsaturated fatty acids;
hsa01212: fatty acid metabolism.
